# How do stand age and site quality shape productivity of Chinese fir plantations via stand structural pathways?

**DOI:** 10.3389/fpls.2026.1783629

**Published:** 2026-04-01

**Authors:** Lijie Wang, Yuanyuan Han, Honggang Sun, Jianfeng Zhang

**Affiliations:** 1 Research Institute of Subtropical Forestry, Chinese Academy of Forestry, Hangzhou, China; 2 Long-term Research Base of National Forestry and Grassland Administration on Forest Vegetation Restoration in Degraded Red Soil Area of Jiangxi, College of Forestry, Jiangxi Agricultural University, Nanchang, China

**Keywords:** *Cunninghamia lanceolata*, productivity, site index, stand age, stand structure, thinning

## Abstract

Stand productivity in plantation forests arises from the interaction among developmental stage, site potential, and structural organization, yet the pathways through which stand age and site quality regulate productivity remain insufficiently clarified in Chinese fir plantations. We examined pure *Cunninghamia lanceolata* plantations across four stand ages (5, 15, 20, and 30 years; 24 plots) in northern subtropical China. Productivity was quantified as total stand volume (TSV) and mean annual increment (MAI). Structural attributes—including stand density, canopy closure (CC), crown diameter ratio (CDR), and growth dominance coefficient (GDC)—were evaluated using multimodel regression and partial least squares structural equation modeling (PLS-SEM). Commercial thinning occurred only in the 20-year-old stands and was treated as a within-stage structural perturbation. Stand MAI followed a unimodal trajectory, peaking at 15 years, whereas TSV increased cumulatively with age, revealing scale-dependent productivity dynamics. Structural variables markedly enhanced explanatory power beyond stand age and site index. Among them, CDR exhibited the strongest association with MAI, while CC and GDC showed positive but moderate effects. Although stand age retained a significant direct association with productivity in SEM, its independent contribution declined after structural attributes were incorporated, indicating that apparent age effects operate primarily through structural succession rather than chronological accumulation. Thinning-induced density reduction at age 20 did not elevate stand MAI beyond the level observed in 15-year-old stands, suggesting that structural reorganization is developmentally constrained. Overall, stand age defines developmental opportunity, site index constrains growth potential, and stand structure mediates the realization of stand-level productivity, highlighting structural optimization as a key pathway for sustainable plantation management.

## 1 Introduction

Stand structure shapes forest productivity and ecosystem functioning by influencing how light, space and soil resources are captured and allocated among trees (Wang et al., 2025a; Zhang et al., 2024a). Under climate change and sustained timber demand, plantations have become central to both renewable wood supply and regional carbon sinks (Poorter et al., 2015). In southern China, Chinese fir (*Cunninghamia lanceolata*) is a dominant fast-growing plantation species, accounting for approximately 12% of the regional forest area. It contributes substantially to timber production and carbon sequestration (Li et al., 2023a; Liu et al., 2024a; Zhou et al., 2016). Despite intensive management, Chinese fir plantations frequently exhibit growth stagnation, structural degradation and declining productivity (Chen et al., 2023; Wang et al., 2025b). Identifying the structural controls of productivity is therefore important for both ecological understanding and precision silviculture.

A large body of work points to stand density, canopy configuration and size differentiation as core structural drivers of productivity in plantations. Density dynamics and competitive regimes constrain growth and underpin yield–density relationships and self-thinning trajectories (Chivhenge et al., 2024; Long et al., 2024). Structural diversity can also shift the density–growth relationship and the position of the self-thinning line (Pretzsch et al., 2024). In Chinese fir plantations, thinning can redistribute growth among tree sizes and modify size inequality, leading to divergent productivity outcomes even within broadly similar developmental stages (Liang et al., 2023). Observational studies further show that stands of comparable age and site quality can differ markedly in density, canopy structure and size differentiation, with corresponding variation in productivity (Liang et al., 2023; Pretzsch et al., 2024; Wang et al., 2024a). Together, these findings support a multivariate evaluation of stand structure as a proximal driver of productivity variation.

A key challenge is to disentangle direct structural controls from indirect pathways mediated through correlated factors. Multivariate analyses indicate that productivity covaries with both stand structure and climate- or site-related variables (Wang et al., 2021a), yet the direction and strength of structure–productivity relationships are not always consistent across forest types and structural states (Wang et al., 2025a). These inconsistencies suggest that different structural attributes may operate through distinct mechanisms, and that apparent structural effects may reflect confounding with stand age or site quality. Explicit causal frameworks that separate direct from mediated effects are therefore required.

Tree morphology provides mechanistic links between competitive structure and growth performance. Competition indices and remotely sensed data are widely used to characterize competition regimes and their spatial patterns (Versace et al., 2019). At the individual level, the height-to-diameter ratio (HDR) reflects allocation between height growth and radial growth and can indicate competitive stress (Liu et al., 2024b). Crown and canopy structure influence light absorption, light-use efficiency and growth efficiency, and may shift under management interventions (Karamzadeh et al., 2023; Plaga et al., 2024). However, morphological indicators such as HDR and crown diameter ratio (CDR) may covary with other structural metrics (e.g. canopy closure (CC) or density-related variables) and should therefore be tested for incremental explanatory power after accounting for stand age and site effects.

Stand age and site quality jointly define the developmental and environmental context for structure–productivity pathways. Site quality is commonly quantified using the site index (SI) and reflects combined climatic and edaphic constraints on long-term growth potential (Guerra-Hernández et al., 2021; Li et al., 2022a; Subedi et al., 2024; Yue et al., 2024). Site effects can interact with density and competition and thereby influence height–diameter relationships and timber assortment structure (Li et al., 2024; Zhang et al., 2021). Productivity is often summarized using mean annual increment (MAI), a widely used indicator of long-term growth efficiency and biomass accumulation (Tian et al., 2024). Along chronosequences, stand age is associated with forest stability and resilience (Vangi et al., 2024), and it can modify canopy structure and rainfall partitioning, with consequences for within-stand light and moisture conditions (Sadeghi et al., 2024). In plantation systems, plant–soil–microbe interactions may additionally contribute to ecosystem functioning across age sequences (Wang et al., 2022, 2025a). These linkages mean that incomplete control of site heterogeneity can confound age effects and structural effects, limiting inference about causal pathways.

Management context further increases the dynamism of structure–function relationships. Density regulation and thinning can alter crown structure and biomass allocation at broad scales (Li et al., 2022b), and thinning can shift growth partitioning among trees of different sizes, thereby modifying size inequality (Liang et al., 2023). Size inequality and competitive asymmetry can be quantified using the growth dominance coefficient (GDC), which indicates whether growth is dominated by large or small trees (Liang et al., 2023; Pretzsch et al., 2024). Thinning can also induce coupled changes in understory diversity and soil processes (Ding et al., 2023; Liu et al., 2025; Turquin et al., 2025; Wei and Liang, 2021; Yu et al., 2022). LiDAR-based studies further indicate that thinning can affect productivity and its stability by restructuring canopy architecture (Cheng et al., 2025). However, in Chinese fir plantations, management effects are still often evaluated using growth rates or single structural indicators, rather than testing whether thinning reshapes the coupling among structural variables such that structure–productivity pathways become strongly context dependent (Liu et al., 2025).

Here, we investigated Chinese fir plantations in the northern subtropics and developed a multilevel analytical framework integrating stand age, site quality (SI), stand structure and tree morphology to quantify both direct effects and indirect pathways shaping productivity. We asked: (1) which structural and morphological variables explain variation in productivity (MAI) after accounting for stand age and SI? (2) to what extent do structural variables exert direct effects versus indirect effects mediated through canopy and morphological traits? and (3) does thinning modify the strength and organization of structure–productivity pathways?

We hypothesize that: H1, stand structural attributes (stand density and canopy closure) explain significant variation in MAI beyond stand age and SI; H2, size differentiation and tree morphology mediate a substantial portion of structural effects on MAI by reflecting competitive asymmetry and crown space occupation; H3, apparent age effects on MAI operate primarily through structural succession and competition rather than time accumulation alone; and H4, thinning alters pathway strengths by reorganizing density, canopy architecture and size inequality, thereby changing structure–productivity coupling.

## 2 Materials

### 2.1 Study site

**Figure 1:**
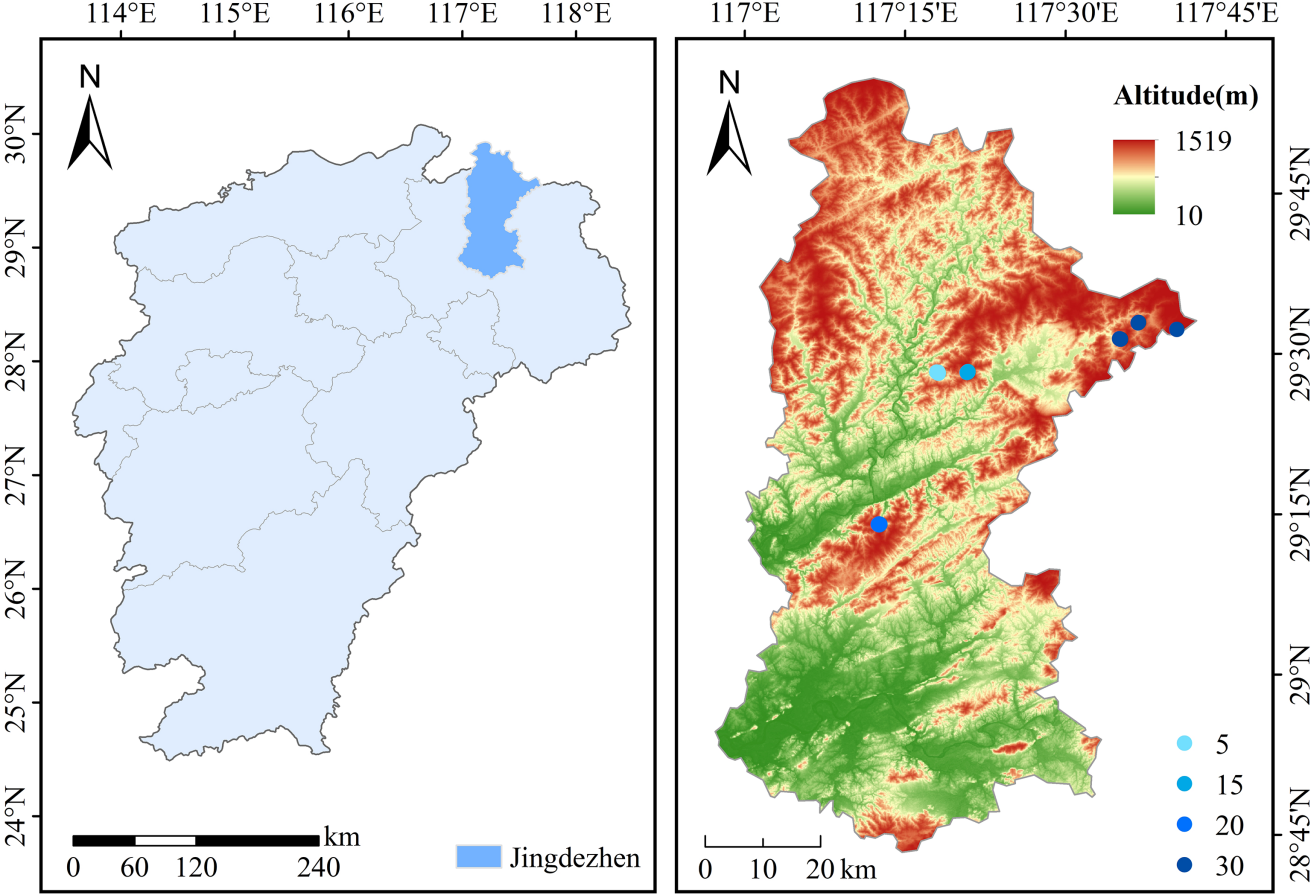
Location of the study area and spatial distribution of long-term forest monitoring plots in Jingdezhen City, Jiangxi Province, China. The main panel shows a topographic and elevation base map of the broader Jingdezhen region, while the inset map (top-left) indicates the geographic position of the study area within Jiangxi Province, with the red bounding box delineating the exact study extent. Sampling plots representing different stand ages (5: 5-year-old stands, 15: 15-year-old stands, 20: 20-year-old stands, and 30: 30-year-old stands) are indicated by distinct symbols. Essential cartographic elements, including a scale bar, graticule (latitude and longitude), and north arrow, are provided. Due to the regional scale of the map and the spatial proximity of replicate plots within the same age class, some plot symbols may appear clustered. Plots of the 5-, 15-, and 20-year-old stands are relatively close to each other (with inter-plot distances greater than 0.5 km), whereas 30-year-old stands are less abundant and therefore more widely spaced, with inter-plot distances generally within 5 km.

This study was conducted at Fengshushan Forest Farm in Jingdezhen, Jiangxi Province, China (29°32′–29°33′ N, 117°36′–117°37′ E; **Figure 1**). The area is characterized by low mountain and hilly terrain (75–320 m) and a subtropical monsoon climate, with a mean annual temperature of 17.8°C and mean annual precipitation of 1650–1850 mm. Soils are deep red soils developed from weathered slate and granite (Alisol, WRB classification), with a soil depth ≥ 60 cm (SChad et al., 2014). Understory vegetation showed clear changes along the stand-age gradient. The 5-year-old stands were dominated by ferns (e.g., *Dicranopteris* spp. and *Woodwardia* spp.). In the 15-year-old stands, shrubs became more prevalent. The thinned 20-year-old stands supported higher understory species richness. In the 30-year-old stands, ferns and shrubs co-occurred and formed a relatively stable community. A detailed species list is provided in **Supplementary Table 1**.

In the 20-year-old stands, commercial thinning was conducted approximately five years prior to the 2022 survey following standard local silvicultural practice. The operation primarily removed suppressed and intermediate trees to reduce stand density and competition intensity, while dominant crop trees were retained. No other stand ages had experienced documented anthropogenic disturbance. Because SI was estimated from the mean height of five dominant trees per plot, and dominant trees were not removed during thinning, SI values were not directly affected by thinning operations.

### 2.2 Experimental design and data collection

Field surveys were conducted in August 2022. Using a space-for-time substitution approach, we established 24 permanent plots (30 m × 20 m; 600 m² per plot, equivalent to 0.06 ha) distributed across four stand ages (5-, 15-, 20-, and 30-year-old stands), with six replicate plots per stand age. This chronosequence design was intended to capture developmental variation in stand structure and productivity. Commercial thinning had been applied exclusively in the 20-year-old stands approximately five years prior to the survey, whereas the 5-, 15-, and 30-year-old stands had not experienced documented anthropogenic disturbance. As a result, stand age and management history are partially confounded at this developmental stage, and thinning is therefore treated as specific to the 20-year-old stands.

Within each plot, all living trees were inventoried. For each individual, diameter at breast height (DBH; cm), total tree height (m), and crown width (m) were measured. These measurements were used to derive stand- and tree-level structural variables, including stand density (trees·ha^-1^), basal area (m^2^· ha^-1^), mean DBH (cm), mean height (m), height-to-diameter ratio (HDR), crown diameter ratio (CDR), growth dominance coefficient (GDC), individual-tree stem volume (MTV; m^3^), mean annual increment (MAI; m^3^·year^-1^), tree-level MAI (MIA-tree; m^3^·year^-1^), and total stand volume (TSV; m^3^).

To further characterize understory responses, detailed shrub and herb surveys were conducted in 12 of the 24 plots. These plots were part of an established long-term ecological monitoring network and were selected because of the availability of comprehensive background data on soil physicochemical properties and microbial communities. Understory surveys were overlaid onto these plots to integrate information on stand structure, nutrient status, and vegetation composition.

In each selected plot, three 5 × 5 m subplots were established to record shrub species identity, abundance, and percentage cover. Within each shrub subplot, one 1 × 1 m quadrat was used to survey herb species identity, abundance, and percentage cover. Shrub layer richness (SLR) and herb layer richness (HLR) were calculated as the total number of shrub and herb species recorded per plot, respectively. Shrub layer cover (SLC, %) and herb layer cover (HLC, %) were visually estimated as the proportional ground area covered by shrubs and herbs within each plot. Total understory cover (TUC, %) was calculated as the combined proportional cover of shrub and herb layers at the plot level. Canopy closure (CC, %) was visually estimated in each plot as the proportion of ground area vertically projected by tree crowns. All understory variables were averaged at the plot level prior to statistical analyses. Stand structural and productivity metrics were derived from the complete tree inventory conducted across all 24 plots.

To quantify variation in site quality, the height of five dominant trees was measured in each plot, and site index (SI) was estimated using the national site index table for Chinese fir (Liu et al., 1982). SI was subsequently included as a continuous covariate in statistical analyses to account for variation in site-related growth potential.

### 2.3 Data processing and analysis

Because thinning occurred only in the 20-year-old stands, its effects were interpreted within that developmental stage rather than as independent treatment effects across stand ages (SA). SI, as described in Section 2.2, was used to represent site quality in subsequent analyses.

Based on the complete tree inventory and subplot surveys, three categories of variables were derived for subsequent analyses: stand growth and productivity metrics, stand structural and competition metrics, and understory vegetation metrics.

#### 2.3.1 Stand growth and productivity metrics

Mean DBH and mean height: Mean DBH (cm) and mean height (m) were calculated as the arithmetic means of DBH and height, respectively, across all living trees within each plot.

Basal area: first, the basal area of each tree was calculated as the cross-sectional stem area at breast height. Plot-level basal area was then obtained by summing individual-tree basal areas within each plot and scaling to a per-hectare basis, as shown in Equation 1:

(1) $$G=\frac{\sum_{i=1}^{n}\frac{\pi }{4}\times {\left(D_{i}\right)}^{2}}{A}\times 10^{-4}$$

where *G* is basal area per hectare (m^2^·ha^-1^); *D_i_*is the DBH (cm); n is the total number of measured trees in the plot; and *A* is the plot area (ha).

Stand volume: first, individual-tree stem volume was estimated using an empirical two-variable volume equation developed for Chinese fir (Equation 2) (Duan et al., 2016). Stand volume was calculated by summing the individual-tree volumes within each plot.

(2) $$MTV=0.000058777042D^{1.9699831}\times H^{0.89646157}$$

where *MTV is* the stem volume of an individual Chinese fir tree (m^3^), *D* is the DBH (cm), and *H* is tree height (m).

Stand volume was then calculated according to Equation 3.

(3) $$TSV=\sum v_{i}$$

where *TSV*is total stand volume within the plot (m ^3^), *v_i_*is the individual-tree volume (m³).

Mean annual increment (MAI) at the stand level was calculated for each stand age by dividing total stand volume (TSV) by stand age (years), providing an estimate of average annual stand productivity (m^3^·year^-1^). In addition, tree-level mean annual increment (MAI-tree) was calculated by dividing individual tree volume by stand age, yielding average annual growth per tree (m^3^·year^-1^).

#### 2.3.2 Stand structure and competition metrics

Stand density: stand density was defined as the number of living trees per unit area (trees·ha^-1^).;

Growth dominance coefficient (GDC) was calculated to quantify size-dependent growth partitioning within a stand using Equation 4 (Binkley, 2004; Binkley et al., 2006):

(4) $$GDC=1-\sum_{i=1}^{n}(d_{i}-d_{i-1})\left(v_{i}-v_{i+1}\right)$$

where *i* denotes the relative rank (position) of a tree, with trees ordered by diameter at breast height (DBH) from largest to smallest; *d_i_*and *v_i_*represent the cumulative proportions of total stand volume contributed by trees ranked from the largest down to the individual. Boundary conditions are defined as *d_0_* = 0 and *v_i+1_* = 0. Positive GDC (ranging from −1 to 1) values indicate that larger trees contribute disproportionately more to stand growth than expected from their structural representation (growth dominance), whereas negative values indicate a relatively greater contribution of smaller trees (reverse dominance). Values close to zero reflect proportional growth partitioning across tree sizes.

Mean height-to-diameter ratio (HDR): the arithmetic mean of the ratio of tree height to DBH across all trees within each plot (King, 1990).

Mean crown diameter ratio (CDR): the arithmetic mean of the ratio of crown width to DBH across all trees within each plot. CDR is used as an index of horizontal canopy space occupation relative to stem size, reflecting how efficiently canopy space is utilized per unit of structural investment. Unlike absolute crown size, CDR emphasizes proportional allocation to lateral crown expansion and thus captures an important dimension of canopy spatial organization under competitive conditions (Plaga et al., 2024).

#### 2.3.3 Statistical analyses

All statistical analyses were performed in R (v4.3.2). Differences in stand structural attributes, productivity metrics, and understory variables among stand-age classes were evaluated using one-way analysis of variance (ANOVA) when assumptions of normality and homogeneity of variances were satisfied. Normality and homoscedasticity were assessed using Shapiro–Wilk and Levene’s tests, respectively. When ANOVA assumptions were met, *post hoc* comparisons were conducted using Tukey’s honestly significant difference (HSD) test. For variables violating parametric assumptions, non-parametric Kruskal–Wallis tests were applied, followed by pairwise Wilcoxon rank-sum tests with Bonferroni correction. Statistical significance was set at α = 0.05.

To evaluate the independent effects of stand age (SA) and site index (SI) on total stand volume (TSV), analysis of covariance (ANCOVA) was conducted. In this model, SA was treated as a categorical fixed factor representing the four stand-age classes (5-, 15-, 20-, and 30-year-old stands), and SI was included as a continuous covariate. Statistical significance of main effects was assessed using F-tests at α = 0.05.

Pearson correlation coefficients were calculated between stand structural metrics (SD, BA, HDR, CDR, GDC, and SI) and productivity metrics (TSV, MTV, and MAI) to characterize bivariate relationships and provide an empirical basis for subsequent multivariate analyses.

To identify structural variables with strong explanatory power for productivity while accounting for stand age and site quality, multivariable linear regression models were fitted with MAI as the focal response variable. SA and SI were included as control variables, and stand structural and competition-related metrics were treated as candidate predictors. Prior to model fitting, multicollinearity among predictors was assessed using variance inflation factors (VIF). Severe collinearity was assumed when VIF > 10, and highly collinear variables were excluded based on ecological interpretability. After screening, SD, CC, CDR, and GDC were retained as structural predictors. All continuous variables were standardized (Z-scores) to facilitate comparison of relative effect sizes.

Given the relatively small sample size and correlations among structural variables, regression coefficients were interpreted primarily in terms of direction and relative contribution rather than precise causal estimates. A set of ecologically interpretable candidate models was constructed and compared using Akaike’s information criterion (AIC) and Bayesian information criterion (BIC) to balance explanatory power and model parsimony. The final model was selected based on minimum information criteria and ecological interpretability.

Building on the regression results, partial least squares structural equation modeling (PLS-SEM) was applied as a complementary analytical framework to disentangle direct and indirect pathways linking stand age, site quality, stand structure, and productivity. In the PLS-SEM, MAI served as the endogenous productivity variable; SA and SI were specified as exogenous variables; and SD, CC, CDR, and GDC were treated as mediating structural variables within a hypothesized path model. All variables were Z-score standardized prior to modeling. A reflective measurement model (Mode A) was used, and the significance of path coefficients and indirect effects was assessed via bootstrapping with 1000 resamples. Model explanatory power was evaluated using coefficients of determination (R²) for endogenous variables, and standardized path coefficients were used to interpret structural pathways influencing productivity.

Understory vegetation data were Z-score standardized prior to principal component analysis (PCA). Differences in understory community composition between thinned and unthinned plots were evaluated using permutational multivariate analysis of variance (PERMANOVA) with 999 permutations. As understory surveys were conducted in a subset of plots, these analyses were interpreted as exploratory rather than fully representative of all stand ages.

## 3 Results

### 3.1 Stand age-related variation in structural and productivity attributes

**Figure 2:**
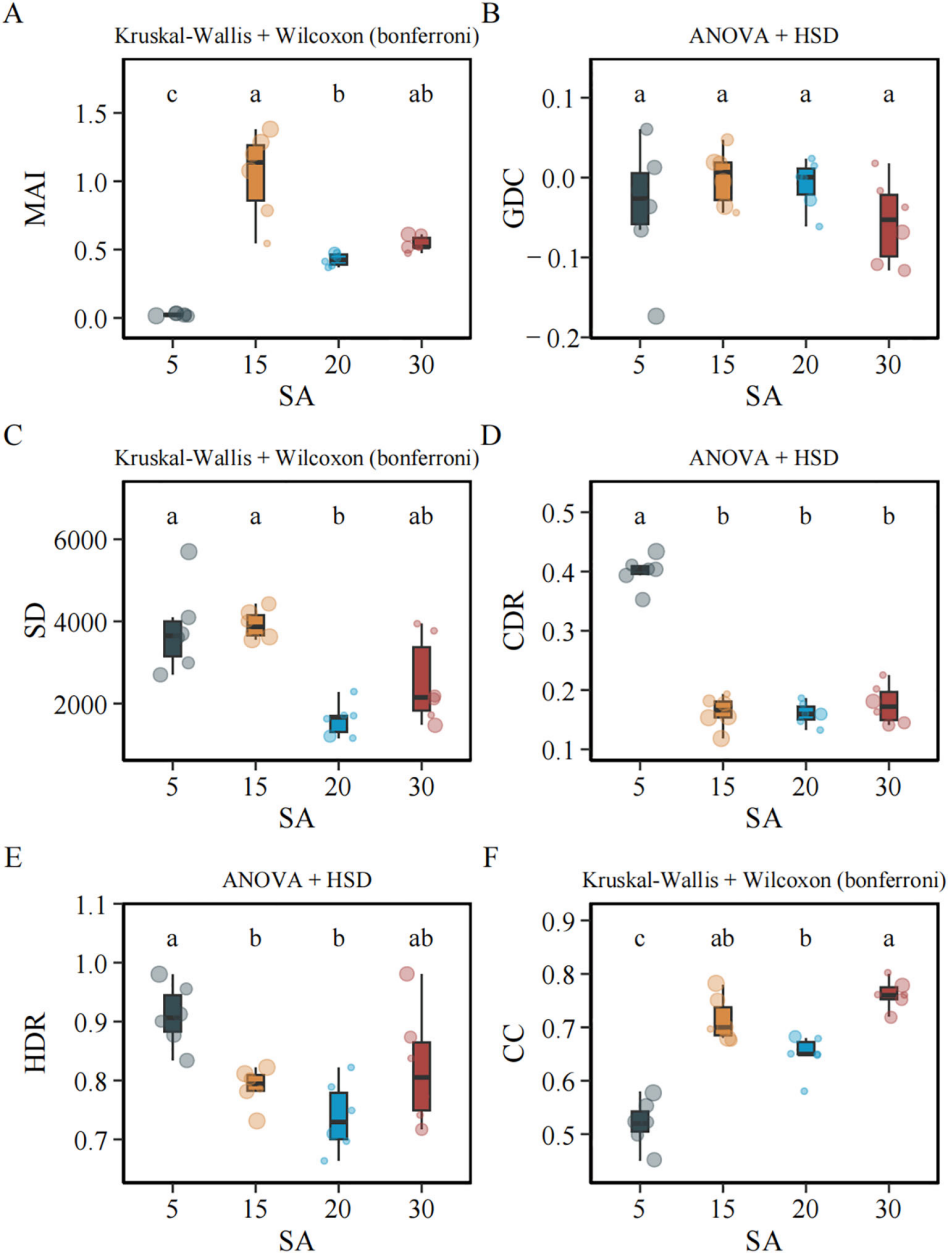
Stand age effects on growth, structural, and morphological attributes in Chinese fir plantations. Boxes represent the interquartile range (IQR; 25th–75th percentiles), horizontal lines indicate medians, and whiskers extend to 1.5 × IQR; points beyond whiskers are plotted as outliers. Overlaid scatter points represent plot-level site index values, with larger point size indicating higher site index. Triangular points denote thinned stands at 20 years of age. Different lowercase letters indicate significant differences among stand ages based on Kruskal–Wallis tests with Bonferroni correction **(A, C, E)** or one-way ANOVA followed by Tukey’s HSD **(B, D, F)**. Groups sharing the same letter are not significantly different (*p* < 0.05). MAI, mean annual increment; GDC, growth dominance coefficient; CDR, crown diameter ratio; HDR, height–diameter ratio.

Chinese fir plantations exhibited pronounced differences in structural, growth, and morphological attributes across stand ages (**Table 1**; **Figure 2**; **Table 2**; *p* < 0.05).

**Table 1:** Stand structural attributes, productivity metrics, and understory vegetation characteristics across four stand ages in Chinese fir plantations (mean ± SE).

Variable	5 yr	15 yr	20 yr	30 yr
SI	16 ± 1a	16 ± 1a	12 ± 0ab	13 ± 1b
SD(trees·ha^-1^)	3800 ± 432	3925 ± 142	1611 ± 168	2539 ± 431
CC(%)	0.52 ± 0.02c	0.72 ± 0.02a	0.65 ± 0.02b	0.76 ± 0.01a
DBH(cm)	3.88 ± 0.16b	12.04 ± 0.58a	13.56 ± 0.68a	14.25 ± 0.87a
Height(m)	3.21 ± 0.07b	9.23 ± 0.43a	9.72 ± 0.33a	11.34 ± 1.22a
BA(m²·ha^-1^)	5.74 ± 0.73c	47.96 ± 4.53a	24.24 ± 0.86b	42.11 ± 1.62a
TSV(m³·ha^-1^)	0.12 ± 0.02c	15.68 ± 1.96a	8.14 ± 0.49b	15.91 ± 0.78a
MAI(m³·ha^-1^·year^-1^)	0.024 ± 0.004c	1.046 ± 0.131a	0.425 ± 0.019b	0.542 ± 0.023b
MAI-tree (m³·year^-1^)	0.0007 ± 0.0001b	0.0044 ± 0.0005a	0.0046 ± 0.0005a	0.0041 ± 0.0007a
MTV(m³)	0.003 ± 0.001c	0.067 ± 0.009b	0.090 ± 0.012ab	0.123 ± 0.023a
GDC	-0.036 ± 0.033a	-0.000 ± 0.014a	-0.008 ± 0.013a	-0.055 ± 0.022a
HDR	0.91 ± 0.02a	0.79 ± 0.01b	0.74 ± 0.02b	0.82 ± 0.04ab
CDR	0.40 ± 0.01a	0.16 ± 0.01b	0.16 ± 0.01b	0.18 ± 0.01b
TUC(%)	13.87 ± 1.54c	20.07 ± 0.67b	27.90 ± 1.42a	16.60 ± 0.84bc
SSR(count)	10 ± 2a	8 ± 1a	11 ± 1a	10 ± 1a
HSR(count)	6 ± 1a	1 ± 0a	3 ± 1a	5 ± 2a
SLC(%)	10.30 ± 0.84b	8.15 ± 0.98b	15.93 ± 0.65a	7.27 ± 0.29b
HLC(%)	4.80 ± 0.82c	16.42 ± 0.79ab	21.2 ± 1.45a	14.45 ± 1.80b

Stand structural and growth variables (e.g., DBH, BA, TSV, MAI, HDR, CDR) were measured across all 24 plots (four stand ages × six replicates). Understory vegetation variables (e.g., shrub and herb species richness, layer coverages) were surveyed in 12 long-term ecological monitoring plots (three per stand age), originally established to represent gradients in site index and stand development. The 20-year-old stands were commercially thinned approximately five years prior to sampling, whereas the 5-, 15-, and 30-year-old stands had not undergone thinning before sampling. Different letters indicate significant differences among stand ages based on *post hoc* multiple comparisons (Tukey’s HSD following one-way ANOVA for normally distributed variables, and Dunn’s test following Kruskal–Wallis tests for non-normally distributed variables; *p* < 0.05). SI, Site Index; SD, Stand Density; CC, Canopy Closure; DBH, Diameter at Breast Height; BA, Basal Area; TSV, Total Stand Volume; MAI, Mean Annual Increment; MAI-tree, Tree-level Mean Annual Increment; MTV, Mean Tree Volume; GDC, Growth Dominance Coefficient; HDR, Height–Diameter Ratio; CDR, Crown–Diameter Ratio; TUC, Total Understory Coverage; SSR, Shrub Species Richness; HSR, Herb Species Richness; SLC, Shrub Layer Coverage; HLC, Herb Layer Coverage.

**Table 2:** ANCOVA evaluating the effects of stand age and site index on total stand volume in Chinese fir plantations.

Source of variation	Df	Sum Sq	Mean Sq	*F*-value	*P*-value
SI	1	11.1	11.1	3.784	0.067
SA	3	1083.9	361.3	123.025	< 0.001
Residuals	19	55.8	2.9		

ANCOVA was used to assess the independent and combined effects of stand age (SA) and site index (SI) on total stand volume (TSV). SI was included as a continuous covariate, whereas SA was treated as a categorical fixed factor representing four stand-age classes (5-, 15-, 20-, and 30-year-old stands). Significant effects were determined using F-tests (α = 0.05). SA, Stand Age; SI, Site Index; Df, degrees of freedom; Sum Sq, sum of squares; Mean Sq, mean square.

SI showed limited variation among stand ages, with only minor differences detected. Values were generally similar in 5- and 15-year-old stands, slightly lower in 20-year-old stands, and moderately higher in 30-year-old stands, although differences were small in magnitude. SD varied significantly among stand ages. Densities were highest in 15-year-old stands and remained high in 5-year-old stands, were substantially reduced in 20-year-old stands, and were intermediate in 30-year-old stands. CC also differed significantly. It was lowest in 5-year-old stands, increased markedly in 15-year-old stands, declined in 20-year-old stands, and reached high values again in 30-year-old stands. DBH and tree height both increased significantly with stand age. DBH and height were lowest in 5-year-old stands and significantly greater in 15-, 20-, and 30-year-old stands, with no significant differences among these older age classes. BA differed significantly among stand ages. BA was lowest in 5-year-old stands, increased substantially in 20-year-old stands, and reached its highest values in 15- and 30-year-old stands, although the 15-year-old stands showed the numerically highest mean. TSV followed a similar but not identical pattern. TSV was lowest in 5-year-old stands, increased in 20-year-old stands, and was highest in 15- and 30-year-old stands, with no significant difference between these two older age classes. MAI exhibited a unimodal pattern. MAI was lowest in 5-year-old stands, peaked in 15-year-old stands, and declined in 20- and 30-year-old stands, which did not differ significantly from each other. At the individual-tree scale, MAI-tree differed significantly among stand ages. MAI-tree was significantly lower in 5-year-old stands than in 15-, 20-, and 30-year-old stands, whereas no significant differences were observed among these three older age classes. MTV increased significantly with stand age, reaching the highest values in 30-year-old stands. Tree-level morphological traits also varied significantly. HDR was highest in 5-year-old stands, significantly exceeding values in 15- and 20-year-old stands, while 30-year-old stands showed intermediate values. CDR was significantly higher in 5-year-old stands than in older stands. In contrast, GDC did not differ significantly among stand ages.

Analysis of covariance (ANCOVA) was performed using TSV as the response variable and SA and SI as explanatory variables (**Table 2**). SA had a significant effect on TSV (F = 123.025, *p* < 0.001), whereas SI was not statistically significant (F = 3.784, p = 0.067).

Understory vegetation attributes, including TUC, SSR, HLR, SLC, and HLC, also differed significantly among stand ages (**Table 1**; *p<* 0.05).

### 3.2 Correlations between stand structure and productivity metrics

**Figure 3:**
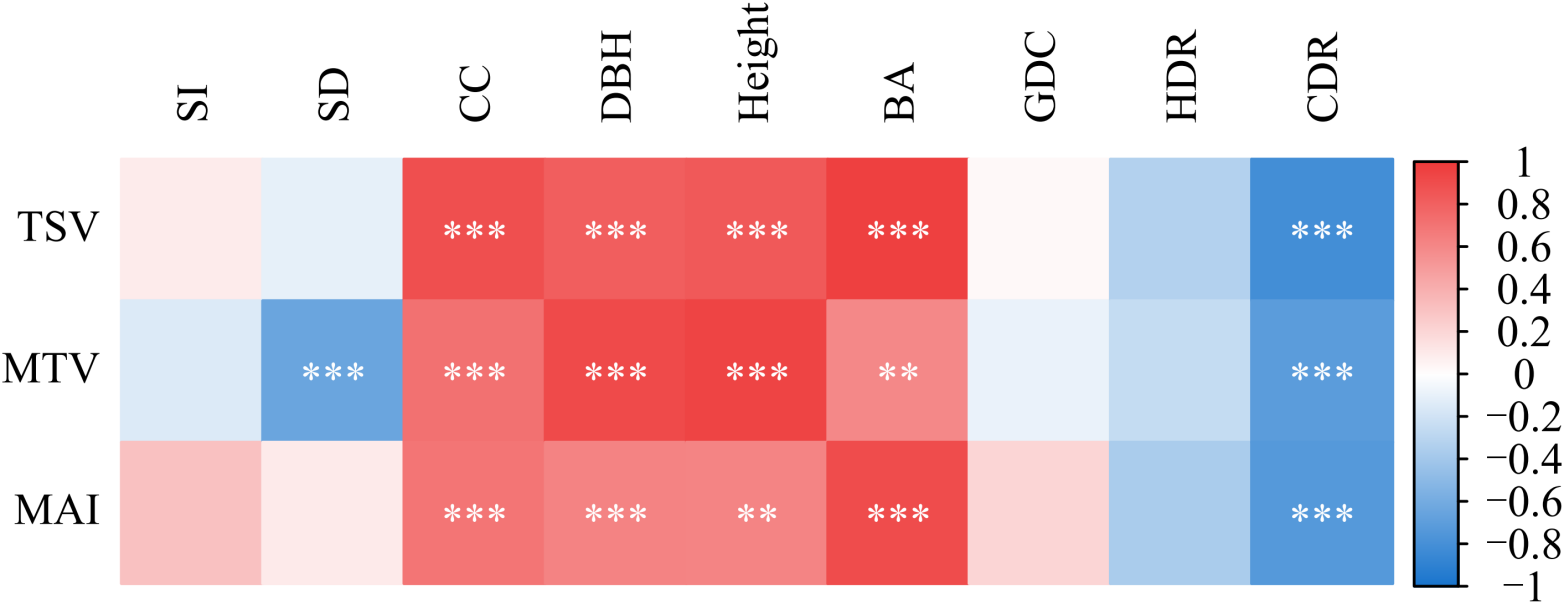
Pearson correlation matrix showing relationships between stand structural attributes and productivity metrics in Chinese fir plantations. The heatmap displays Pearson correlation coefficients between three productivity indices—total stand volume (TSV), mean tree volume (MTV) and mean annual increment (MAI)—and key stand structural variables. Color intensity represents correlation strength (red = positive, blue = negative). Asterisks indicate significance levels (**p < 0.01; ***p < 0.001). SI, Site Index; SD, Stand Density; CC, Canopy Closure; DBH, Mean Diameter at Breast Height; BA, Basal Area; GDC, Growth Dominance Coefficient; HDR, Height–Diameter Ratio; CDR, Crown–Diameter Ratio.

Correlation analysis revealed clear association patterns between stand structural attributes and productivity metrics in Chinese fir plantations (**Figure 3**). The strength of the relationships varied widely, with R² values ranging from 0.001 to 0.939.

TSV, MTV, and MAI were significantly and positively correlated with DBH, Height, BA, and CC (*p* < 0.01 or *p* < 0.001). Among these relationships, the strongest association was observed between TSV and BA (R² = 0.939).

Density-related attributes showed contrasting relationships with productivity metrics. MTV was significantly negatively correlated with SD (*p* < 0.001). In addition, TSV, MTV, and MAI were all significantly negatively correlated with CDR (*p* < 0.001).

In contrast, correlations between productivity metrics and SI, GDC, and HDR were generally weak or not statistically significant (*p* > 0.05).

### 3.3 Drivers of stand productivity and their relative importance

Using multimodel comparison and multivariable regression, we evaluated the relationships between stand structural attributes and mean annual increment (MAI) and quantified their relative contributions to variation in productivity (**Tables 3**, **4**; **Supplementary Tables 2** and **3**; **Supplementary Figure 1**).

**Table 3:** Comparison of multiple regression models explaining variation in mean annual increment in Chinese fir plantations.

Model	Formula	AIC	BIC	ΔAIC	R^2^	Adj.R^2^	Akaike weigh
M1	MAI ~ SA	26.36	29.89	58.82	0.12	0.08	0.56
M2	MAI ~ SI	26.99	30.52	59.45	0.10	0.06	0.44
M3	MAI ~ SA + SI	18.50	23.21	50.96	0.42	0.36	0.00066
M4	MAI ~ SA + SI + SD + CC	4.88	11.95	37.34	0.72	0.66	0.0000071
M5	MAI ~ SD + CC + CDR + GDC	-1.45	5.62	31.01	0.79	0.76	0.0000001
M6	MAI ~ SA + CC + SD + CDR + GDC	-9.90	-1.66	22.56	0.87	0.82	0.000000004
M7	MAI ~ SA + SI + SD + CC + CDR	-18.999	-10.74	13.48	0.90	0.88	~0
M8	MAI ~ SI + CC + SD + CDR + GDC	-31.98	-23.74	0.48	0.94	0.92	~0
M9	MAI ~ SA + SI + SD + CC + CDR + GDC	-32.46	-23.04	0	0.95	0.93	~0

Multiple linear regression models were constructed to evaluate the effects of stand structural attributes on MAI. Candidate models were built by sequentially adding explanatory variables, including SA, SI, SD, CC, CDR, and GDC. Model performance was assessed using Akaike Information Criterion (AIC), Bayesian Information Criterion (BIC), coefficient of determination (R²), and adjusted R². Lower AIC and BIC values indicate better model fit. ΔAIC and Akaike weights were calculated to evaluate relative model support. SA, Stand Age; SI, Site Index; SD, Stand Density; CC, Canopy Closure; CDR, Crown–Diameter Ratio; GDC, Growth Dominance Coefficient; MAI, Mean Annual Increment.

**Table 4:** Regression coefficients and relative contributions of stand structural variables explaining mean annual increment in Chinese fir plantations.

Variable	β	SE	t	p	Std_β	Partial_R^2^
(Intercept)	-1.0949	0.3694	-2.9639	0.0087		
CDR	-2.6764	0.4112	-6.5082	< 0.001	-0.7069	0.7136
SI	0.0771	0.014	5.5047	< 0.001	0.4327	0.6406
GDC	1.8711	0.4769	3.9239	0.0011	0.2534	0.4753
CC	1.7822	0.6396	2.7863	0.0127	0.4398	0.3135
SD	< 0.001	< 0.001	1.5639	0.1363	0.144	0.1258
SA	-0.0099	0.0073	-1.3606	0.1914	-0.2258	0.0982

This table presents the results of the optimal multiple regression model evaluating the effects of stand structural variables on mean annual increment (MAI). Reported statistics include regression coefficients, standard errors, test statistics and significance levels for each explanatory variable. Partial coefficients of determination (Partial R²) were calculated to quantify the independent contribution of each variable after accounting for the effects of other predictors in the model. Statistical significance was evaluated using t-tests at α = 0.05. CDR, Crown–Diameter Ratio; SI, Site Index; GDC, Growth Dominance Coefficient; CC, Canopy Closure; SD, Stand Density; SA, Stand Age.

Model comparison showed a progressive increase in explanatory power with the inclusion of structural predictors (**Table 3**). Single-predictor models including only SA or SI explained limited variation in MAI (R^2^ = 0.12 and 0.10, respectively). The combined model including SA and SI improved model fit (R^2^ = 0.42). Additional inclusion of SD and CC further increased explanatory power (R^2^ = 0.72). Models incorporating CDR and GDC showed substantially higher explanatory performance (R^2^≥0.90).

Among the candidate models, M9 had the lowest AIC (−32.46; ΔAIC = 0; Akaike weight = 0.56), indicating the strongest support among the candidate models, although M8 (ΔAIC = 0.48; weight = 0.44) received comparable support. All other models had ΔAIC values greater than 10 and negligible Akaike weights.

In the full model (M9), SI (β = 0.4327, *p* < 0.001), CC (β = 0.4398, p = 0.0127), and GDC (β = 0.2534, p = 0.0011) were positively associated with MAI, whereas CDR was negatively associated with MAI (β = −0.7069, *p* < 0.001). SA (p = 0.19) and SD (p = 0.14) were not statistically significant predictors.

Partial R^2^ values showed that CDR explained the largest unique proportion of variance in MAI (partial R^2^ = 0.7136), followed by SI (0.6406), GDC (0.4753), and CC (0.3135). The unique contributions of SA (0.0982) and SD (0.1258) were comparatively small (**Table 4**). Crown diameter ratio accounted for the largest unique proportion of explained variation in MAI, followed by canopy closure and site index, whereas the unique contributions of stand age and stand density were comparatively small. Collectively, these results indicate that variation in stand productivity across the studied plantations is more closely associated with canopy structural attributes and indicators of individual growth capacity than with stand age or density alone.

### 3.4 Structural equation modeling of mechanisms underlying stand productivity

**Figure 4:**
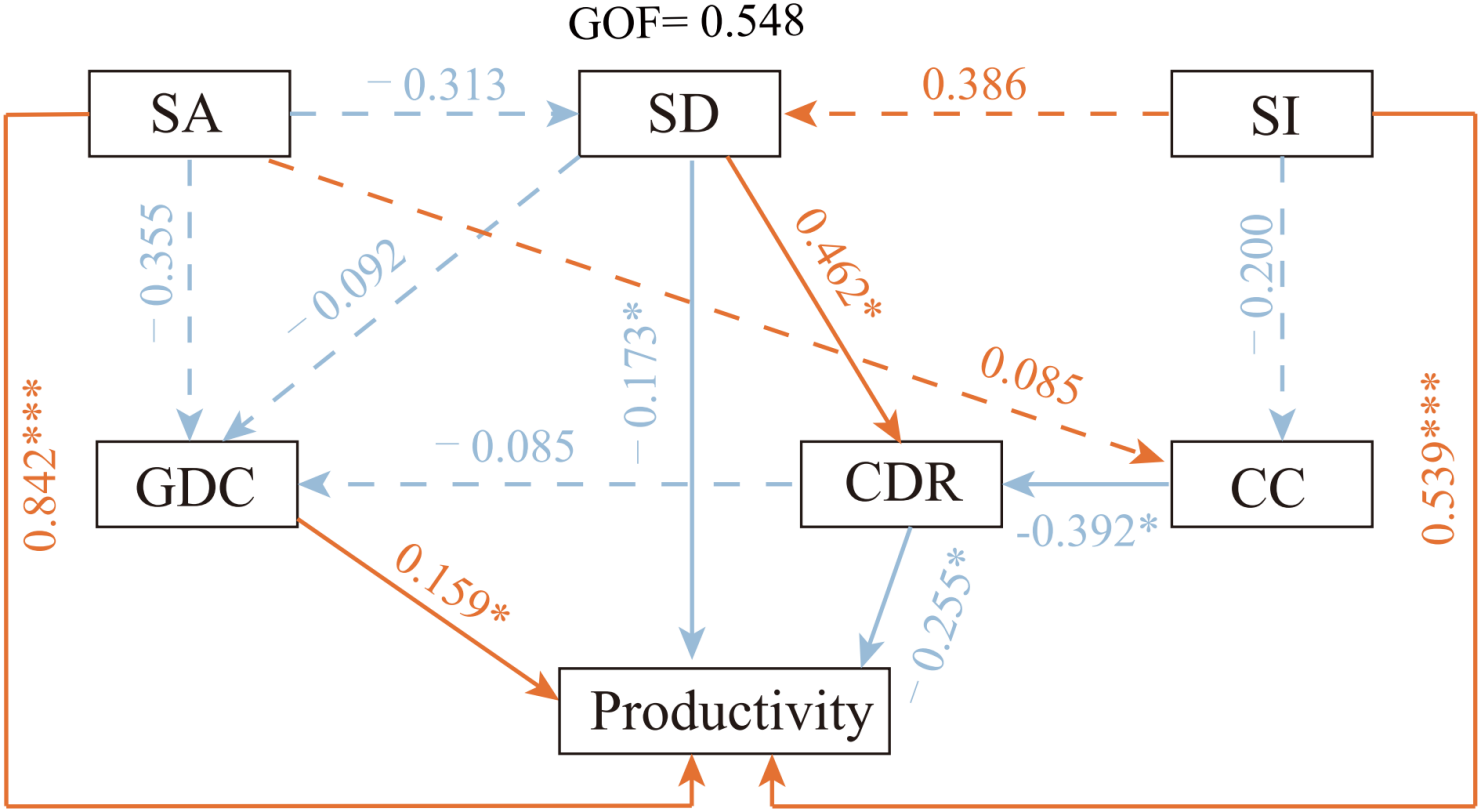
Partial least squares structural equation model (PLS-SEM) illustrating the direct and indirect pathways through which stand age, site index, and stand structural attributes regulate productivity in Chinese fir plantations. Solid arrows indicate significant pathways (∗ *p* < 0.05), whereas dashed arrows denote non-significant relationships. Numbers adjacent to arrows represent standardized path coefficients (β). Stand age is interpreted as a developmental stage indicator integrating cumulative competition history and structural succession, rather than as elapsed time alone. Stand density, canopy closure, crown diameter ratio, and growth dominance coefficient act as mediating structural variables linking developmental stage and site quality to realized productivity. Asterisks indicate significance levels (∗ *p* < 0.05; ∗∗∗ *p* < 0.001).

Partial least squares structural equation modeling (PLS-SEM) was used to quantify the direct and indirect pathways through which SA, SI, and stand structural attributes were associated with productivity in Chinese fir plantations (**Figure 4**; **Supplementary Figure 2**; **Supplementary Table 4**). Overall model fit was acceptable (GOF = 0.548), and the coefficient of determination for Productivity was high (R² = 0.941), indicating that the model captured a substantial proportion of the observed variation in stand productivity.

SA and SI both exhibited significant positive direct associations with Productivity. The standardized path coefficient from SA to Productivity was 0.842 (*p* < 0.001), while that from SI to Productivity was 0.539 (*p* < 0.001), with stand age showing the strongest standardized effect among all exogenous variables. In contrast, the effects of stand structural attributes on Productivity varied in both direction and magnitude. SD and CDR were negatively associated with Productivity (β = −0.174, p = 0.049; β = −0.255, p = 0.033, respectively), whereas GDC showed a significant positive association (β = 0.159, p = 0.036). The direct effect of CC on Productivity was negative but not statistically significant (β = −0.075, p = 0.097).

Within the structural subnetwork, SD was positively associated with CDR (β = 0.462, p = 0.016), while CC was negatively associated with CDR (β = −0.392, p = 0.038). Neither SD nor CDR showed significant relationships with GDC (SD → GDC: p = 0.706; CDR → GDC: p = 0.726). Stand age did not exert significant effects on SD or CC (β = −0.313 and 0.085, respectively), whereas the effect of SI on SD was marginal (β = 0.386) and its effect on CC was not significant (β = −0.200).

Notably, stand age explained only a limited proportion of variation in individual structural attributes, while maintaining a strong association with overall productivity.

### 3.5 Understory vegetation patterns across stand developmental stages

**Figure 5:**
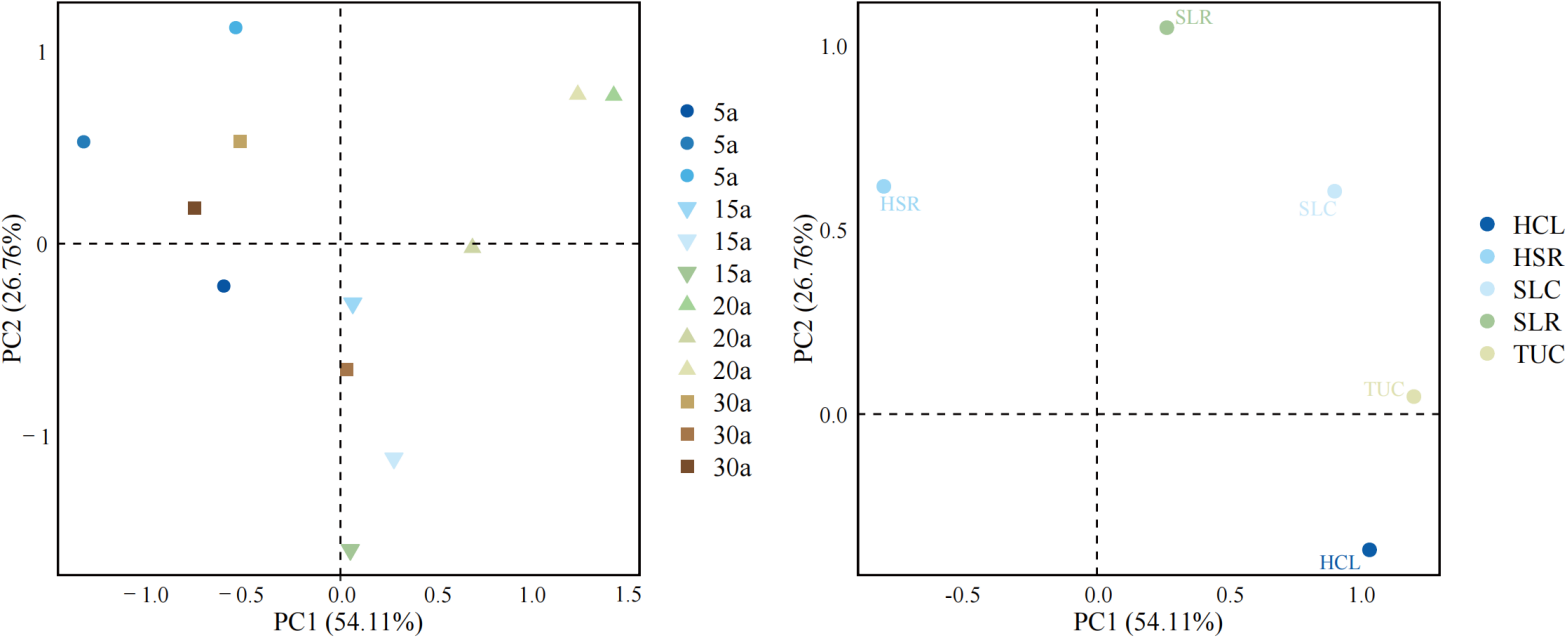
Principal component analysis (PCA) of understory vegetation structure across four stand ages in Chinese fir plantations. PCA was performed on shrub species richness (SSR), herb species richness (HSR), shrub layer coverage (SLC), herb layer coverage (HLC), and total understory coverage (TUC). The left panel displays the ordination of plots from four stand ages (5-, 15-, 20-, and 30-year-old stands), while the right panel shows the loadings of the five understory variables.

Principal component analysis (PCA) of understory vegetation attributes showed that the first two axes together explained 80.87% of the total variance (PC1 = 54.11%, PC2 = 26.76%; **Figure 5**). PC1 was primarily associated with variation in understory cover, with HLC and TUC showing strong positive loadings. Plots from 20-year-old stands were clearly shifted toward higher PC1 scores.

PC2 mainly reflected variation in species richness, with SLR and HSR contributing most strongly. Plots from 5- and 30-year-old stands were separated along PC2.

## 4 Discussion

Using stand structure, growth, and productivity data from 24 Chinese fir plantation plots, we quantified the multi-pathway mechanisms through which stand age, site quality, and stand structure jointly shape productivity. In contrast to studies that emphasize single-factor effects or bivariate associations, our analyses explicitly separated direct effects from indirect effects transmitted through structural mediators, highlighting the central regulatory role of stand structure in translating potential productivity into realized productivity.

### 4.1 Stand age and site quality: a productivity baseline and an upper constraint

Stand age defines the developmental context within which stand productivity unfolds, whereas site index reflects underlying growth potential. However, stand age should be interpreted not as a direct causal driver but as an integrative indicator of accumulated developmental processes—including competition, mortality, spatial redistribution, and canopy reorganization—through which structural state, competitive history, and management-induced reorganization ultimately determine how this potential is realized.

This view is consistent with evidence that stand development alters tree resource-use strategies and allocation patterns beyond a simple function of elapsed time (Zhang et al., 2024b). Stand age and density interact to influence tree growth, water use, and soil carbon processes (Chen et al., 2025; Sun et al., 2023), while competition-induced mortality progressively reshapes the density–growth relationship through self-thinning (Long et al., 2025; Pretzsch et al., 2023; Zhao et al., 2025).

The chronosequence further revealed a scale-dependent pattern of productivity. Stand-level MAI reached its maximum in the 15-year-old stands, whereas TSV increased cumulatively with age, and MAI-tree remained relatively similar among the 15-, 20-, and 30-year-old stands. These contrasting trajectories emphasize that productivity metrics capture different dimensions of stand development. MAI reflects per-area growth efficiency, TSV cumulative biomass retention, and MAI-tree individual-tree performance. The relative stability of MAI-tree across later stages suggests that variation in stand-level MAI primarily arises from differences in density, canopy organization, and size structure rather than intrinsic changes in tree-level growth. Similar scale-dependent relationships between size inequality and stand-level production have been documented in Chinese fir plantations (Liang et al., 2023), supporting the interpretation that stand structure mediates how individual growth is translated into area-based productivity.

Management legacy must be interpreted cautiously. In this study, commercial thinning was conducted exclusively in the 20-year-old stands and is therefore treated as a within-stage structural perturbation rather than as a general thinning effect applicable across all stand ages. Although structural intervention occurred at age 20, stand-level MAI remained below the peak observed in 15-year-old stands. This suggests that density reduction alone does not guarantee immediate enhancement of per-area productivity. Instead, productivity responses at age 20 likely reflect thinning-induced structural reorganization operating within that specific developmental stage. Comparable findings in other plantation systems indicate that growth and carbon sequestration responses depend jointly on density, rotation stage, and stand structural context (Bai and Ding, 2024; Ni et al., 2022; Wei et al., 2024; Manrique-Alba et al., 2025).

Physiological and functional characteristics of Chinese fir provide additional context for these structural dynamics. Chinese fir may be vulnerable to prolonged or seasonal drought and may not rely solely on xylem embolism resistance to maintain hydraulic function (Ren et al., 2024). Canopy architecture and leaf retention traits respond strongly to light environment (Zhou et al., 2025), and root spatial distribution varies with topography and resource gradients, influencing water and nutrient acquisition efficiency (Li et al., 2025a). Under high density, simultaneous light limitation and belowground competition may restrict the expression of site advantages.

The distinction between potential and realized productivity offers a useful interpretative framework (Liu et al., 2021; Yan et al., 2023). Site index defines a potential upper boundary of growth, but whether this potential is expressed depends on density and structural configuration. Previous studies suggest that stand density and site quality can interact to influence nutrient status and growth environment (Sun et al., 2025), and that adjustments in stand structure may facilitate the expression of site-related growth potential (Gabira et al., 2023; Li et al., 2025b, 2023b). In our study, this pattern suggests that structural reorganization following thinning did not immediately translate into enhanced realized productivity.

Overall, these findings indicate that stand age structures the temporal trajectory of stand-level productivity, site index reflects background growth potential, and stand structure mediates the translation of individual-tree growth into per-area production. Together, they support a hierarchical interpretation in which developmental stage constrains structural configuration, and structure in turn regulates productivity realization. Because this study is based on a space-for-time substitution design, unmeasured historical differences among stands cannot be entirely excluded; therefore, these relationships should be interpreted as structured associations rather than definitive causal effects.

### 4.2 Structural pathways regulating stand-level productivity

Building on Section 4.2, stand-level productivity (stand MAI and TSV) was interpreted in relation to structural regulation. Because commercial thinning occurred only in the 20-year-old stands, it is treated as a within-stage structural perturbation, and this section focuses on the broader ecological spillover of that reorganization. Accordingly, the SEM pathways were discussed as structured associations consistent with the conceptual model, not as definitive causal effects.

#### 4.2.1 Stand density as a quantitative constraint

Stand density acted primarily as a quantitative constraint regulating how individual-tree growth was aggregated into per-area production. Classical yield–density theory and self-thinning relationships provide the ecological basis for this interpretation (Drew and Flewelling, 1977; Weller, 1987; Mrad et al., 2020) and density management diagrams illustrate how crowding structures growth trajectories in even-aged stands (Newton, 2021). Empirical syntheses further show that density effects vary with developmental stage and structural adjustment (West, 2024; Yue et al., 2020). In the present chronosequence, density reduction at age 20 did not correspond to higher stand MAI than that observed in the 15-year-old stands. This pattern indicates that density alone does not determine productivity. Instead, realized production depends on how canopy organization and size structure adjust following changes in crowding. Experimental evidence similarly demonstrates that competition manipulation alters growth responses in a context-dependent manner (Donfack et al., 2023).

#### 4.2.2 Crown form, canopy organization, and efficiency regulation

Beyond quantitative crowding, canopy spatial organization emerged as a key regulator of production efficiency. CDR was closely aligned with stand MAI and reflected horizontal crown expansion relative to stem size. Greater lateral crown expansion can increase crown overlap and intensify light competition among neighboring trees, which may reduce overall canopy space-use efficiency at the stand scale (Pretzsch, 2014). In contrast, more compact crown configurations can promote clearer vertical layering and more effective spatial partitioning within the canopy, thereby enhancing stand-level resource-use efficiency (Jucker et al., 2015; Xu et al., 2024). Studies in Chinese fir and mixed forests show that crown dimensions respond predictably to competition and reflect canopy packing processes (Wang et al., 2021b; Hou and Chai, 2022). Broader syntheses indicate that canopy spatial configuration often explains productivity variation more effectively than individual-tree traits alone (Forrester et al., 2018; Wang et al., 2024b). Analyses comparing structural and site contributions in Chinese fir systems similarly highlight the importance of stand structure in regulating productivity outcomes (Li et al., 2023b; Wang et al., 2023). Together, these findings support the interpretation that crown form captures a structural dimension closely linked to how individual growth is translated into stand-level MAI and accumulated as TSV. Although several morphological metrics were evaluated, only CDR retained consistent explanatory relevance in the multivariate framework.

Not all structure-related metrics played equally strong mediating roles. Although the GDC provided insight into size-dependent growth partitioning, its explained variance in the SEM was low. It therefore appeared to provide complementary information on allocation dynamics rather than acting as a dominant regulator of stand-level productivity under the studied conditions.

Taken together, the SEM pathways supported a hierarchical interpretation in which stand structure mediated how developmental context and site potential were expressed as stand-level productivity (stand MAI and TSV). Importantly, because commercial thinning occurred only in the 20-year-old stands, the structural configuration observed at that stage reflected a within-stage reorganization rather than a general thinning effect across ages. This framing provided a basis to examine whether the same structural reorganization that was associated with stand-level productivity also coincided with broader ecological responses, particularly in the understory.

### 4.3 Thinning legacy and ecosystem spillover effects

Building on the structural pathways discussed above, we interpret the age-20 patterns as consequences of thinning-induced reorganization. Within this structural framework, commercial thinning occurred only in the 20-year-old stands and therefore represented a within-stage structural perturbation specific to that developmental stage. Accordingly, this section interprets the age-20 patterns as consequences of thinning-induced structural reorganization and its broader ecological spillover beyond the overstory.

Across plantation systems, thinning primarily modifies stand structural states and competitive dynamics, with growth responses depending on context and time since intervention rather than showing uniform immediate gains in per-area production (Jian et al., 2025; Svystun et al., 2025; Reid et al., 2025; Ding and Zang, 2021; Kong et al., 2023). Consistent with this view, thinning in the present chronosequence reorganized competition and resource capture patterns, which coincided with differences in stand-level production (stand MAI and TSV) observed at age 20.

Importantly, the ecological consequences of this structural reorganization were not confined to the overstory. In the thinned 20-year-old stands, understory attributes differed markedly relative to other ages, with higher total understory cover and shifts in shrub-layer development and richness under a more open canopy environment (**Table 1**). These responses are consistent with evidence that canopy opening alters light and near-ground microclimate, promoting understory recovery and compositional adjustment (Yang et al., 2023; Molina et al., 2022; He et al., 2025). In turn, understory development may influence soil biochemical properties, nutrient cycling, microbial function, and litter decomposition pathways, particularly under disturbance and reorganization contexts (Molina et al., 2022; Lu et al., 2023; Wu et al., 2025).

From a management ecology perspective, these patterns support a cascading pathway whereby overstory structural adjustment influences understory and soil processes, rather than a simple “thinning increases productivity” narrative. Because the chronosequence design cannot fully separate age effects from management legacy at age 20, these results should be interpreted as structured associations tied to observed stand conditions.

Finally, by increasing vertical and horizontal heterogeneity in vegetation structure, thinning-associated reorganization may have implications for broader ecosystem functioning beyond stand-level production. Structural and compositional shifts in understory communities can modify microsite heterogeneity and habitat complexity, which may contribute to multifunctionality and stability in forest systems (Forrester and Bauhus, 2016; Cheng et al., 2024). More generally, spatial and temporal variability in site quality reinforces the need to interpret structural interventions within developmental context rather than as universally transferable prescriptions (Skovsgaard and Vanclay, 2013). The age-20 case illustrates how structure-oriented management may influence multiple ecosystem components simultaneously, aligning with close-to-nature silvicultural perspectives and restoration-oriented thinning approaches (Bauhus et al., 2013; Case et al., 2023; Šilinskas et al., 2024).

In summary, within this study’s design, thinning at age 20 is best interpreted as a within-stage structural intervention that reorganized canopy conditions and competitive environments, with measurable spillover to understory communities and belowground processes, rather than as a general yield-enhancement effect (Bauhus et al., 2013; He et al., 2025; Wu et al., 2025).

### 4.4 Study limitations

Our study has several limitations. First, the space-for-time substitution design assumes comparability among stands of different ages. Although plots were selected to minimize site variation, residual microsite heterogeneity and unmeasured historical differences cannot be excluded. Thus, the reported relationships should be interpreted as structured associations consistent with the conceptual framework rather than as definitive causal effects. Second, commercial thinning occurred only in the 20-year-old stands and was therefore confounded with stand age at that stage. Because unthinned 20-year-old stands and thinned older stands were not included, management history cannot be fully disentangled from age-related effects. Accordingly, productivity responses observed at age 20 should be interpreted as thinning-related structural reorganization within that developmental stage, not as a general thinning effect across stand ages. Third, all plots were located within a single region, which strengthens internal consistency but limits broader generalization. Replicated studies across wider environmental gradients and long-term permanent plots would improve external validity and mechanistic inference.

## 5 Conclusions

This study demonstrates that stand-level productivity in Chinese fir plantations, expressed as MAI and TSV, reflects the joint influence of developmental stage and site quality, but is most directly associated with stand structural organization. Stand age and site index define the temporal and environmental context of growth potential, whereas stand structure mediates how that potential is translated into realized productivity. In particular, stand density and canopy configuration represent key structural dimensions associated with variation in MAI and TSV across developmental stages.

Within this framework, thinning in the 20-year-old stands is best interpreted as a within-stage structural intervention rather than as a generalized yield-enhancement treatment. Productivity patterns at that stage were associated with thinning-induced reorganization of stand density and canopy structure, rather than with a direct thinning effect per se. Structural reorganization was accompanied by marked understory responses, suggesting that structure-oriented management can influence multiple ecosystem components beyond the overstory. Collectively, these findings underscore the central role of stand structure in mediating productivity dynamics and highlight structural optimization as a promising pathway for improving productivity and ecosystem functioning in Chinese fir plantations.

## Data Availability

The raw data supporting the conclusions of this article will be made available by the authors, without undue reservation.
