# Supplementary material for: How do stand age and site quality shape productivity of Chinese fir plantations via stand structural pathways?
Source: Front Plant Sci. 2026 Apr 1;17:1783629. doi: 10.3389/fpls.2026.1783629 (PMC13079582; doi:10.3389/fpls.2026.1783629)
Supplement: Supplementary file 1 [file Table1.docx]

Supplementary materials

**Table S1.** Dominant understory plant species in Chinese fir plantations across different stand ages.

| Stand age year | Dominant herb layer | Dominant shrub laye |
| --- | --- | --- |
| 5 | *Dicranopterispedate,*  *Woodwardia japonica* | *Smilax china*, *Itea chinensis*,*Eurya nitida*,*Adinandramillettii* |
| 15 | *Woodwardia japonica* | *Loropetalumchinense*,*Iteachinensis*,*Camelliacuspidate*,*Lasianthus japonicus* |
| 20 | *Woodwardia japonica* | *Itea chinensis*, *Camellia pubicosta*, *Rubus buergeri*, *Weigela florida*,*Callicarpa japonica* |
| 30 | *Woodwardia japonica*, *Dicranopterispedate*, *Dryopteris erythrosora*, *Dryopteris dickinsii* | *Lindera aggregate*,*Loropetalumchinense*,*Maesa japonica*,*Callicarpa americana* |

**Table S2.** Regression coefficients and significance levels of stand structural variables in the best‐supported multiple regression model.

| Variable | Standardized coefficient (Std. β) | β | SE | t | p | VIF |
| --- | --- | --- | --- | --- | --- | --- |
| (Intercept) | - | 0 | 0.0531 | 0 | 1 |  |
| SA | -0.2258 | -0.2258 | 0.1659 | -1.3606 | 0.1914 | 9.3581 |
| SI | 0.4327 | 0.4327 | 0.0786 | 5.5047 | 0 | 2.1002 |
| SD | 0.144 | 0.144 | 0.092 | 1.5639 | 0.1363 | 2.8794 |
| CC | 0.4398 | 0.4398 | 0.1578 | 2.7863 | 0.0127 | 8.4661 |
| CDR | -0.7069 | -0.7069 | 0.1086 | -6.5082 | 0 | 4.009 |
| GDC | 0.2534 | 0.2534 | 0.0646 | 3.9239 | 0.0011 | 1.4174 |

**Table S3.** Summary of overall model fit statistics and significance tests.

| Metric | Value |
| --- | --- |
| R² | 0.95 |
| Adjusted R² | 0.9323 |
| Sample size(n=24) | 24 |
| F-statistic | 53.81 |
| p-value | ＜0.001 |

**Table S4.** Standardized path coefficients and explained variance of the PLS-SEM

Panel A. Structural paths

| **Endogenous variable** | **Path** | **Std. β** | **SE** | **t** | **p** | **Supported** |
| --- | --- | --- | --- | --- | --- | --- |
| **SD** | SA → SD | −0.313 | 0.199 | −1.58 | 0.129 | No |
|  | SI → SD | 0.386 | 0.199 | 1.95 | 0.065 | Marginal |
| **CC** | SA → CC | 0.085 | 0.241 | 0.35 | 0.727 | No |
|  | SI → CC | −0.200 | 0.241 | −0.83 | 0.416 | No |
| **CDR** | SD → CDR | 0.462 | 0.178 | 2.61 | 0.016 | Yes |
|  | CC → CDR | −0.392 | 0.178 | −2.21 | 0.038 | Yes |
| **GDC** | SD → GDC | −0.092 | 0.240 | −0.38 | 0.706 | No |
|  | CDR → GDC | −0.085 | 0.240 | −0.36 | 0.726 | No |
| **Productivity** | SA→ Productivity | 0.842 | 0.115 | 7.30 | <0.001 | Yes |
|  | SI→ Productivity | 0.539 | 0.087 | 6.24 | <0.001 | Yes |
|  | SD→ Productivity | −0.174 | 0.082 | −2.12 | 0.049 | Yes |
|  | CC → Productivity | −0.075 | 0.072 | −1.05 | 0.097 | No |
|  | CDR→ Productivity | −0.255 | 0.110 | −2.32 | 0.033 | Yes |
|  | GDC → Productivity | 0.159 | 0.070 | 2.28 | 0.036 | Yes |

Panel B. Explained variance of endogenous variables

| Endogenous variable | R² |
| --- | --- |
| SD | 0.364 |
| CC | 0.064 |
| CDR | 0.342 |
| GDC | 0.022 |
| Productivity | 0.941 |


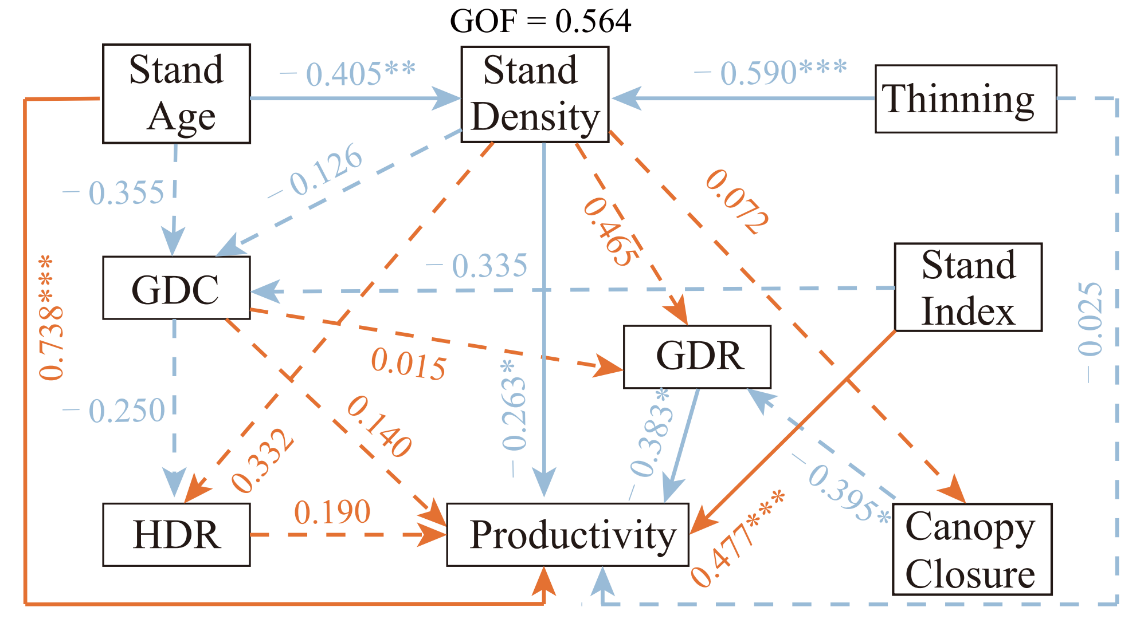


Figure S1. Alternative PLS-SEM including thinning as an explicit management indicator. Thinning was coded as a binary variable (thinned vs. unthinned). Because commercial thinning occurred exclusively in the 20-year-old and was therefore fully nested within a single developmental stage, this model is presented as a robustness comparison rather than for primary causal inference. Despite this limitation, the alternative model yields qualitatively consistent results in highlighting the central role of stand density and canopy structure in mediating productivity responses, supporting the structure-driven interpretation presented in the main text.
